# Microencapsulation of fish oil by spray-drying using two different wall materials: a comparison

**DOI:** 10.1590/1414-431X2025e14007

**Published:** 2025-06-16

**Authors:** S.C.B. Pinto, A.M. Kluczkovski, H.B. Lemos, V.G. Torres, A.V.B. Jesus, T.P. Souza

**Affiliations:** 1 Faculdade de Ciências Farmacêuticas, Universidade Federal do Amazonas, Manaus, AM, Brasil

**Keywords:** Maltodextrin, Microencapsulation, Oil, Skimmed milk, Spray-dryer

## Abstract

Oils and fats have a wide range of health benefits, especially those that have a high concentration of polyunsaturated fatty acids, like fish oils. Unfortunately, oils are highly susceptible to heat, light, and oxygen degradation, causing them to lose the qualities that would make them interesting for the public. With this in mind, this study compared two methods of spray-drying, a technique that helps preserve the stability of oils in storage for longer periods of time. Emulsions made with skimmed milk powder and with maltodextrin and whey protein hydrolysate were spray-dried, resulting in 2 different microencapsulated fish oils. They were compared regarding encapsulation efficiency, water activity, moisture content, and peroxide index. The skimmed milk showed better results, with a 28.17% lower peroxide index than the non-encapsulated oil and lower water activity and moisture content compared to the emulsion using maltodextrin (2.690±0.19% *vs* 6.747±0.29% moisture content and 0.376±0.013 *vs* 0.554±0.006 water activity). Since skimmed milk powder is rather cheap, it is ideal for spray-drying, a simple and fast technique. In this way, fish oil can be safely microencapsulated in powder form, lasting longer than the oil capsules currently available, since the oil is protected from light, temperature, moisture, and oxidation. In addition, the oily odor is masked, making it more appealing to the consumer, and it may be combined with other powders, like vitamins and minerals, which opens up new possibilities for the production of supplements.

## Introduction

Oils and fats are formed by glycerol and chains of fatty acids, which can be obtained from plant and animal materials. Linoleic and linolenic acids are examples of essential fatty acids that are involved in many cellular functions and can be found in plant and fish oils. Polyunsaturated fatty acids (PUFAs) are found in fish oil extracts, and docosahexaenoic acid (DHA) and eicosapentaenoic acid (EPA), which are long-chain omega-3 fatty acids, are the predominant PUFAs derived from fish oils (1).

Studies have demonstrated the benefits of ingesting these compounds in the prevention and treatment of cancer, inflammation, asthma, rheumatoid arthritis, osteoporosis, and allergies, as well as improving the development and functioning of the central nervous system. Omega-3 fish oils are claimed to have several benefits, including prevention of cardiovascular disease, reduction of cognitive decline, and better management of inflammatory diseases (2,3).

However, oils are susceptible to oxidative degradation, forming peroxides and other chemicals. These chemicals are responsible for undesirable aromas in oils, and some of them can be toxic, causing health problems for the consumer. In addition, oils have low solubility in water, making them difficult to use in the development of new products. To overcome this problem, studies have shown that the stability of oils can be maintained by encapsulation (4).

One method of oil encapsulation is spray-drying, which has a short processing time. Spray-drying encapsulated hydrophilic bioactive compounds such as oils enhances the bioactive stability of these products during storage, protecting them from light, heat, and oxygen. In addition, this technique helps to mask the taste and odor of fish oils, thus making them more attractive to consumers (5,6).

Microencapsulation of ingredients involves the formation of a microcapsule generally composed of a polymer that acts as a physical barrier against the external environment and consequently, against oxidation and relative humidity, for example. This polymeric film breaks down under certain conditions, which allows the substance to be released at the ideal time or place. In the case of oils, the spray-drying process can also produce high-quality powders. Even though the temperature of the air used in this process is high, the temperature inside the microcapsule is much lower as the drying takes place at high speed and in a short time (7,8).

With that in mind, the objective of this study was to compare 2 spray-drying encapsulation methods using different materials and to select the best method to be used in the development of products, such as supplements, to be offered to consumers as new ways to improve health.

## Material and Methods

### Oil acquisition

The oil was extracted from fish samples obtained from the local market in Manaus, Amazonas. The fish were cut into small pieces and put in a pan with water no hotter than 90°C. This allowed the fat to melt and be extracted without any issue. This was then centrifuged at 1677 *g* for 10 min at 25°C and the oil was collected for use.

### Emulsion and encapsulation by spray-drying

The emulsions were prepared with 10% fish oil and distilled water as the aqueous phase. Whey protein hydrolysate (WPH) and skimmed milk powder were used as emulsifying agents in the following concentrations: emulsion 1 (E1): 80 g of WPH was added to the aqueous phase and stirred using a laboratory mixer at 500 rpm for 2 h at room temperature. Next, 80 g of oil was added to the emulsion, with the help of a mixer at 24,000 rpm for 5 min. After that, 160 g of maltodextrin was added and mixed for about 5 h at 500 rpm to form the “wall” of the microcapsules. Then, atomization by spray drying was started (2). Emulsion 2 (E2): 240 g of skimmed milk powder was added to the aqueous phase and stirred at 500 rpm for 1 h. This solution was stored at 4°C for 10 h to ensure complete hydration. Eighty grams of oil was slowly added to this solution and the emulsion was then homogenized at 24,000 rpm for 5 min (9).

Both emulsions had the ratio of 10:30:60 for oil, wall material (WPH, maltodextrine, and skimmed milk powder), and water. The emulsions were then spray-dried at inlet temperatures of 140°C, nominal aspirator rate of 3.81×10^-3^, and spray flow rate of 40 mL/min (2,9).

### Encapsulation efficiency

This test determines how good the encapsulation process was, where higher results mean that more oil was effectively encapsulated, and less oil was left on the surface of the powder. To determine the encapsulation efficiency, the amount of surface oil was measured. For that, 15 mL hexane was added to 2 g of capsule followed by shaking for 2 min at room temperature. The suspension was then filtered through filter paper and the residue was then rinsed 3 times with 20 mL hexane. The filtrate was transferred to an oven at 75°C for 5 h to evaporate the hexane. To determine the surface oil, the difference between the initial and final weight of the dried filtrate was calculated, and the encapsulation efficiency was obtained with the following equation:

$$Encapsulation\;efficiency=\;\frac{\left(Total\;oil-Surface\;oil\right)}{Total\;oil}\;x\;100\;$$ (Eq. 1)

The tests were made in triplicate with each emulsion (9).

To further analyze the efficiency, the capsules were observed through a scanning electron microscope (SEM). For this process, the samples were adhered to carbon tapes on stubs and subjected to metallization with gold (around 15 nm) in the JEOL Smart Coater metallizer with subsequent analysis using the JSM-IT500HR scanning electron microscope from Jeol Ltd. (Japan).

### Peroxide index

The peroxide index is used to determine how “fresh” an oil is, where the higher the peroxide value, the higher the level of degradation of the oil, since peroxide is formed by breaking the bond of triacylglycerols, leaving free radicals that react with oxygen and other chemicals. The oil contained within the microcapsules was extracted using 10 g sodium salicylate and 10 g sodium citrate dissolved separately in reverse osmosis (RO) water, then combined with 18 mL of 1-butanol. The final volume of the solution was 90 mL using RO water to complete it. After that, 10 g of capsule was mixed with 20 mL of the solution at 50°C in a 250 mL Erlenmeyer flask. The mixture was then stirred magnetically in a 70°C water bath for 10 min and centrifuged at 419 *g* for 20 min at 25°C (10).

The extracted oil was then subjected to peroxide index determination according to Association of Official Analytical Chemists (AOAC) (11) methods. Approximately 5 g of fish oil was weighed in a 250 mL Erlenmeyer flask, to which 30 mL of acetic acid-chloroform solution 3:2 v/v was added, and then shaken until the sample dissolved. Then, 0.5 mL of saturated potassium iodide solution was added, and the solution was left to rest away from light for 1 min. Water (30 mL) was added, followed by titration with a previously standardized 0.01 N sodium thiosulfate solution, with constant stirring. The titration continued until the yellow color almost disappeared, when 0.5 mL of indicator starch solution was added, continuing the titration until the blue color completely disappeared. This test was repeated 3 times with each emulsion.

### Moisture content and water activity

The water activity was determined using the Water Activity Meter Dew Point 4TE equipment from AquaLab (USA), by determining the dew point of the sample. Enough sample was added to a capsule to cover its bottom. The capsule was then placed in the water activity meter, the lid was closed, and the vapor equilibrium was awaited. An infrared beam focused on a small mirror determines the precise dew point of the sample. The dew point temperature was then translated into water activity. Analyses were carried out in triplicate (12).

Moisture content (%) was determined using the Gehaka IV3100 Moisture Analyzer equipment (Brazil), which has an infrared halogen lamp. About 2 g of sample was added to the scale, then the lid was closed and the reading began. Sensors are used for correctly reading temperature and moisture content levels. Analyses were carried out in triplicate (13).

## Results and Discussion

Overall, our results pointed favorably towards E2, showing better results regarding encapsulation efficiency, moisture content, water activity, peroxide index, and powder appearance (Table 1).

**Table 1:** Physiochemical properties of microcapsules obtained by the two emulsion methods.

Variables	Emulsion 1	Emulsion 2
Encapsulation efficiency (%)	69.439±0.860	82.148±0.226
Moisture content (%)	6.747±0.29	2.690±0.19
Water activity (%)	0.554±0.006	0.376±0.013
Peroxide index (mEq/kg)	3.806±0.037	3.368±0.048

Whey protein hydrolysate and skimmed milk powder were used as emulsifying agents in emulsion 1 and emulsion 2, respectively. Data are reported as means and SD.

### Encapsulation efficiency and peroxide index

**Figure 1:**
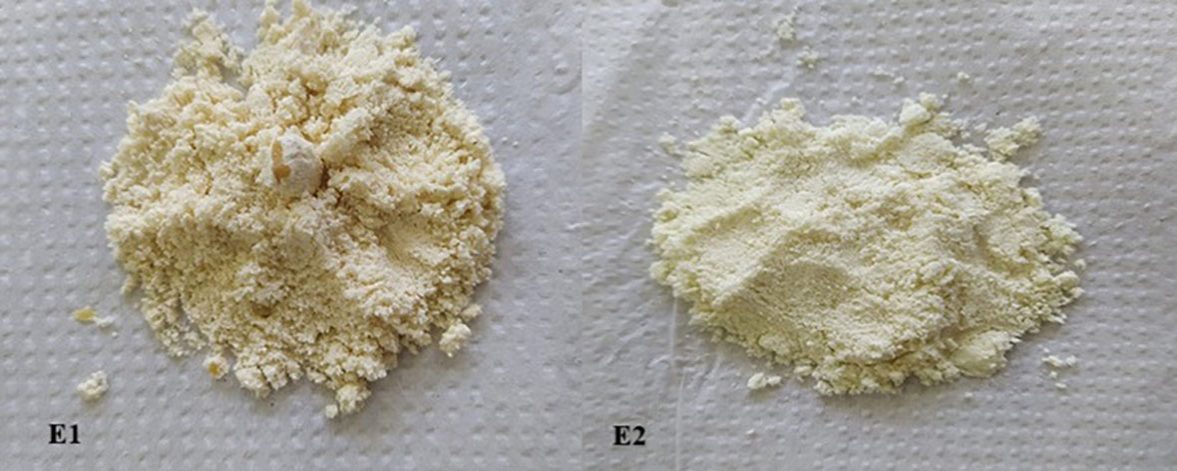
Microcapsules obtained after spray-drying emulsions E1 and E2. E1 presented hard clumps of maltodextrin and E2 was a smoother powder. Whey protein hydrolysate and skimmed milk powder were used as emulsifying agents in E1 and E2, respectively.

**Figure 2:**
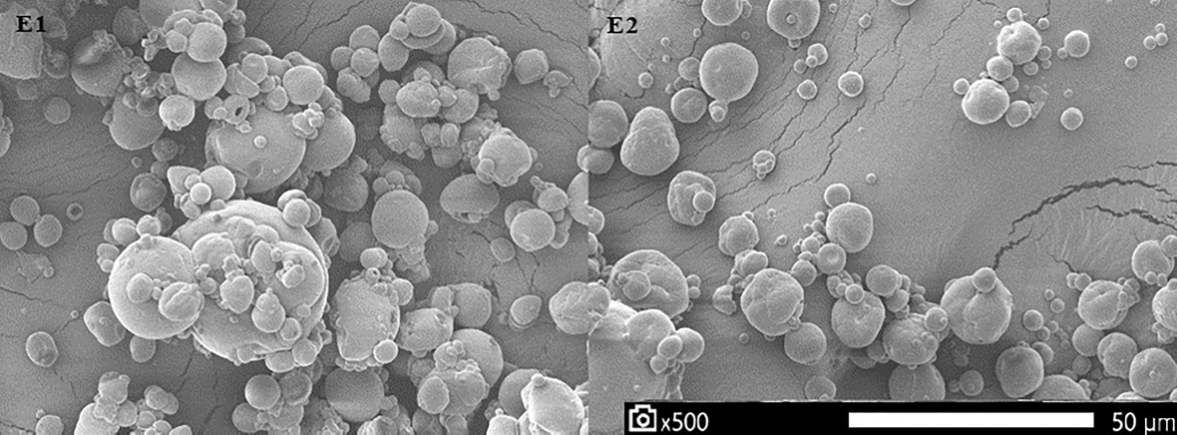
Microcapsules observed using scanning electron microscopy at 500× magnification (scale bar=50 μm). The capsules serve as a barrier, enveloping the oil inside. E1 (left side) had larger capsules, some of them with cracks on the capsule wall and many capsules clumped together. E2 (right side) had smaller capsules that were better spread throughout the area, with little or no cracks on the capsule wall.

When comparing encapsulation efficiency, E2 had better results overall, showing more than 80% efficiency, against a little less than 70% for E1. Also, the product from E1 showed hardened clumps of maltodextrin and WPH, which E2 did not have and which was a lot smoother (Figure 1). When observed through the SEM, E2 also had many more loose capsules compared to E1, where it could be seen that a lot of the capsules were clumped together (Figure 2). When analyzing the powder, E2 was more fluid and free, and the better the powder flows, the easier it is to use.

Other authors evaluated fish oil encapsulation with whey protein and found similar results in the microscopy analysis. It seems that the method provides good encapsulation efficiency and manages to create an acceptable surface of the microcapsule (14). Our peroxide value was actually lower than the one obtained in that study, and our method using skimmed milk had even better results than what was obtained using whey protein and maltodextrin (14).

When comparing the results obtained with skimmed milk, our experiment showed results similar to Aghbashlo et al. (9). The microcapsules obtained were smooth and with little or no cracks in the walls.

Encapsulation efficiency is the percentage of entrapment of a material within a capsule. A high encapsulation efficiency reduces oxidative degradation of the surface of compounds and increases product stability. Oil is a material highly susceptible to oxidative degradation (6,15).

To prepare an encapsulation, 4 steps are necessary: core formation, encapsulant formation, incorporation of core and capsule, and solidification. The core is the primary material that is going to be encapsulated. The encapsulant is used to “trap” the core. Usually, the encapsulant is transformed into a liquid before being used. In both methods used in this study, the encapsulant was first diluted in water. For the incorporation, an aqueous solution and an oil were subjected to mixing at high rpm to prepare an appropriate emulsion for the last step. Solidification is a method used to better handle the product, and it can be done by chemical, physical, or a combination of both methods. The high temperature of the spray-drying process was used as the solidification method in this study (15-17).

Microencapsulation is defined as a method where particles or droplets are enveloped by a coating wall, forming small capsules, where the material you wish to protect is trapped inside. This can be used to protect solids, liquids, and even gases, and to deliver those components. As such, the process of microencapsulation can form a functional wall between the core product and the exterior, avoiding physical and chemical reactions that could spoil the product. Microencapsulation is used to protect bioactive constituents like essential oils, vitamins, and lipids that are chemically unstable, being susceptible to thermal and chemical degradation from extraction to storage. In addition, the process of encapsulation can also help in introducing such components into foods, drinks, and supplements, helping to mask bad taste and/or aroma (5,18,19).

To assess oil degradation, the peroxide index was evaluated and compared between both emulsions and the free oil exposed to the effects of light, temperature, and oxidation. The peroxide index of the free oil was 4.689±0.027 mEq/kg, while E1 had an index of 3.806±0.037 mEq/kg, 18.83% lower, and E2 had an even lower peroxide index, 3.368±0.048 mEq/kg, 28.17% lower. These values point to an increased stability of the oil trapped in a microcapsule.

Lipid oxidation is one of the causes of food spoilage, and it is a greater issue when the food has a high percentage of oils. For this reason, the peroxide index is considered one of the most important criteria for evaluating the quality of food. Oil is decomposed by processes like hydrolysis and oxidation, changing the concentration of hydrogen ions present in the oil. When exposed to heat and light, this process is increased and free fatty acid radicals react with oxygen, forming peroxides. These peroxides can also react with other molecules, creating a chain reaction that forms products like aldehydes and esters, both being volatile, giving a strong odor to the degraded oils (20,21).

Another advantage is the fact that handling powder is a lot easier than handling liquids. Powder can be filled into a hard capsule, a process much easier than the production of soft capsules, which requires more specialized equipment to be made and maintained. A hard capsule can be filled not only with the microencapsulated fish oil as a powder, but also with vitamins and minerals, making it a more complete supplement that can be sold to the consumer (7).

### Moisture content and water activity

E1 showed higher moisture content (6.747±0.29%) and water activity (0.554±0.006) than E2 (2.690±0.19% moisture content and 0.376±0.013 water activity), meaning that E2 was better. Both moisture content and water activity are important parameters of food products. Foods with water activity lower than 0.6 are considered microbiologically stable, meaning a lower chance of contamination by fungus and/or bacteria. This means that both emulsions can be stored for longer periods of time without getting contaminated (22).

Moisture content, as with water activity, is used to measure the stability of powder products, being associated with drying efficiency and storage stability. Microcapsules that present low moisture content are more resistant to mildew and degradation, having increased stability during storage. A moisture content between 4 to 10% is considered adequate for dry foods (23).

Aghbashlo et al. (9) obtained moisture contents ranging from 2.11 to 5.67% when encapsulating fish oil, and Botrel et al. (24) obtained even lower values, with moisture content below 2% when microencapsulating fish oil using soy protein isolate and inulin as wall materials. Our results are in line with those findings. The high temperature of spray-drying decreased the water content of the product.

## Conclusion

The results showed that microencapsulating fish oil was an efficient method for maintaining product stability. Spray-drying of oils from fish, but also of essential oils from plants, opens up new possibilities for the production of new, better, and more stable products not only in the food industry, but also in the medical and pharmaceutical sectors.
